# AutoGIS processing for site selection for solar pond development as efficient water treatment plants in Egypt

**DOI:** 10.1038/s41598-023-44047-0

**Published:** 2023-10-09

**Authors:** Mahmoud Fatehy Altahan, Mohamed Nower

**Affiliations:** 1 Central Laboratory for Environmental Quality Monitoring (CLEQM), National Water Research Center (NWRC), El-Qanater El-Khairia, 13621 Egypt; 2 Water Management Research Institute (WMRI), National Water Research Center (NWRC), El-Qanater El-Khairia, 13621 Egypt

**Keywords:** Climate sciences, Environmental sciences

## Abstract

The increasing demand for renewable and environmentally friendly energy sources is a top priority for many countries around the world. It is obvious that renewable solar energy will help to meet most of the energy demand in the coming years. A solar pond is a huge Salt artificial Lake that serves as a solar energy collection system. However, site selection is a critical factor that affects the effectiveness and lifetime of a solar pond. Here, we present an innovative methodology for site selection based on three environmental factors, including direct solar irradiance (DNI), temperature, and wind speed. Our approach uses Python programming and clustering analysis with several libraries, including Pandas, Geopandas, Rasterio, Osgeo, and Sklearn, to analyse and process data collected over a 30-year period from NASA power. This method was applied to the geographic boundaries of Egypt, but the methods can be applied to any spatial context if the same dataset is available. The results show that Egypt has a potential land area of 500 km^2^ suitable for solar ponds construction along the border with Sudan throughout the year, including 2000 km^2^ in winter (between January and March), 800 km^2^ in spring (between April and June), 900 km^2^ in summer (between July and September), and the largest area of 3700 km^2^ (between October and December), most of which is located in the south of the Eastern Desert and around the Nile River. Notably, the northwestern region, close to the Mediterranean Sea on the border with Libya, exhibits suitability for solar pond development, with consistent performance throughout the year. Our results provide an efficient way for GIS and data processing and could be useful for implementing new software to find the best location for solar ponds development. This could be beneficial for those interested in investing in renewable energy and using solar ponds as an efficient water treatment plant.

## Introduction

The excessive increase in dependence on non-renewable energy sources in recent decades is leading to serious impacts on our environment and planet. Increased energy production and consumption from non-renewable energy sources such as the burning of fossil fuels like oil, coal, and natural gas contribute to the emission of 90% of total global greenhouse gases (GHGs)^1^. GHGs are one of the most important causes of climate change with its severe consequences for the environment and humanity. One of the most important goals of sustainability concepts in recent years has been the search for new renewable energy sources^2^. However, the search for new, powerful, and clean renewable energy sources is ongoing. The availability of renewable energy sources can be divided into four areas: Solar, Geothermal, Wind and Biomass. Of all the available sources, solar radiation is still the most important energy source in the world due to its clean and sustainable nature^3^. One of the most important technologies for storing solar energy is solar pond, which has played a significant role in the field of renewable energy in recent decades^4^.

Solar ponds are referred to as large saltwater lakes that are used to collect and store solar energy and can be used for electricity generation, heating, and other various applications. The technology itself is very simple, highly reliable, cost effective, and produces no emissions. Solar ponds can play an important role in enhancing water quality by providing a sustainable means of treating wastewater and minimizing pollution. As the sun's rays heat the water at the bottom of the pond, the water rises and brings with its nutrients and other pollutants, which are then concentrated in the top layer of the pond. By removing this top layer, solar ponds can effectively treat polluted water and improve water quality^5^. Also, these ponds can be designed to support the growth of beneficial algae and microorganisms, which can help to break down and remove pollutants from the water. Additionally, the use of solar energy in these ponds can help to reduce energy costs and minimize the environmental impact of traditional wastewater treatment methods^6^.

In Egypt, there is a lot of literature on solar ponds used for various purposes. For instance, El-Amin et al. in 2014 described an electricity generation system that uses a solar pond. It is based on the use of solar energy and hydropower to generate electricity in the Qattara depression. A large chimney is located above a solar pond where a thermal gradient has been created to drive air circulation and generate wind that drives a turbine to generate electricity^7^. In 2012, Madkour et al. conducted a study to provide insight into the ecological processes that occur in solar ponds on the far northeast coast of the Sinai. The study provides insight into the composition of phytoplankton in the solar pond, where there are a variety of phytoplankton species in the water. This is attributed to the unique environmental conditions created by the solar pond system. The authors note the importance of their findings for the development of such environmentally friendly salt production systems^8^. While in 2020, Shalaby et al. demonstrated a way to implement a solar pond to recover the brine (high salt concentration) solution out from the Reverse osmosis process. Also, Nower et al. publish 2020 and 2022 Solar pond performance enhances nonconventional water resource availability and implemented solar ponds in Fayoum governorate in Egypt^9,10^.

Solar ponds rely mainly on solar radiation to function, and their location certainly affects their efficiency^11^. Cluster analysis is one of the statistical tools used to group the set of objects to maximize similarity within groups and minimize similarity between groups^12^. In site selection, especially for solar ponds, cluster analysis is used to group spatial data for sites based on the similarity of their solar radiation patterns^13^.

In the last two decades, there are a lot of the literature on the use of geographical information system (GIS) searching for the optimal locations for renewable energy facilities.

For instance, Amjad et al. proposed a method for identifying and evaluating sites for solar farm development in Pakistan using data from GIS and applying density-based clustering techniques. The study focused on clustering areas with the greatest energy potential and excluded data for infrastructure requirements^14^. However, the method presented in the article is so complicated that it is very difficult to be implement by non-specialist users.

In 2022, Garca et al. proposed the use of multiple criteria fuzzy logic-based decision making (MCDM) in combination with GIS data. The method was used to select the optimal location for offshore wind turbines in the Gulf of Maine in the United States. They conclude that using fuzzy GIS -based MCDM can provide a more accurate and reliable selection process^15^. Fuzzy logic is a novel mathematical approach that uses linguistic variables such as “very low”, “low”, “medium”, and “high” instead of the binary logic of “true/false” in traditional data processing. However, the use of fuzzy logic and GIS can make the decision-making process more complicated for non-experts.

Heo et al. described a study that investigates the use of Building Information Modelling (BIM) with overlay data from GIS to determine optimal locations for photovoltaic power plants. The methodology used in this work was based on 3D modelling of potential power plant sites. Site selection was done by grouping areas with similar characteristics such as land availability, solar radiation, and proximity to electrical infrastructure^16^. However, the integration of BIM with GIS is still in the early stages of exploration and has some limitations, such as the limited availability of tools, which makes it difficult for organizations to adopt this approach. In addition, not all data from GIS and BIM can be integrated, so the incompatibility of data leads to inaccurate results in the selection processes^17^.

AutoGIS is a powerful tool that can automate the analysis of geographic information (GIS) so that repetitive tasks can be automated. The tool can provide users with an interface between GIS and users in processing geospatial data. AutoGIS can customize the analysis of GIS data to meet the specific needs of users^18–20^.

Here we report on a study to identify suitable sites for Solar Ponds development, where the automated processing of GIS has proven to be extremely helpful in conducting this type of study. Missing among all the published articles on the site selection process is an implementation of Python programming for AutoGIS processing. Our approach stands out from all the published literature as it provides a quick and easy way for all site selection processes. In doing so, all processes are simulated in a code that is directly usable by non-experts without giving them insight into all the complicated steps. The study aims to use the density-based cluster analysis techniques in Python to implement the automated GIS processing for Solar Ponds development in Egypt with respect to three types of satellite remote sensing data (solar radiation, temperature, and wind speed).

Using the automated GIS processing tools in Python, the density-based clustering algorithms are applied for the first time in this study, especially the DBSCAN (Density-Based Spatial Clustering of Applications and Noise). Our methodology is suitable for spatial data analysis and identification of suitable sites for solar ponds development.

The project includes various phases such as data acquisition, data pre-processing and data analysis using DBSCAN clustering algorithms. Various Python modules were used in the project, highlighting the importance of DBSCAN as an effective tool for identifying optimal locations for solar ponds development and contributing to the development of inclusive and sustainable energy strategies using Egypt as an example.

This work will contribute to the use of such Python libraries for AutoGIS processing in the cartographic representation of water quality patterns. This could support the cartographic representation of data collected by on-site water quality sensors^21–29^ recently reported by our group.

## Results and discussion

As mentioned earlier, solar ponds are innovative technology that can harness the sun's energy to generate electricity and provide heat for a variety of purposes. The design of these ponds is mainly aimed at storing and absorbing large amounts of solar energy, which can then be used in different ways to meet energy needs.

**Figure 1:**
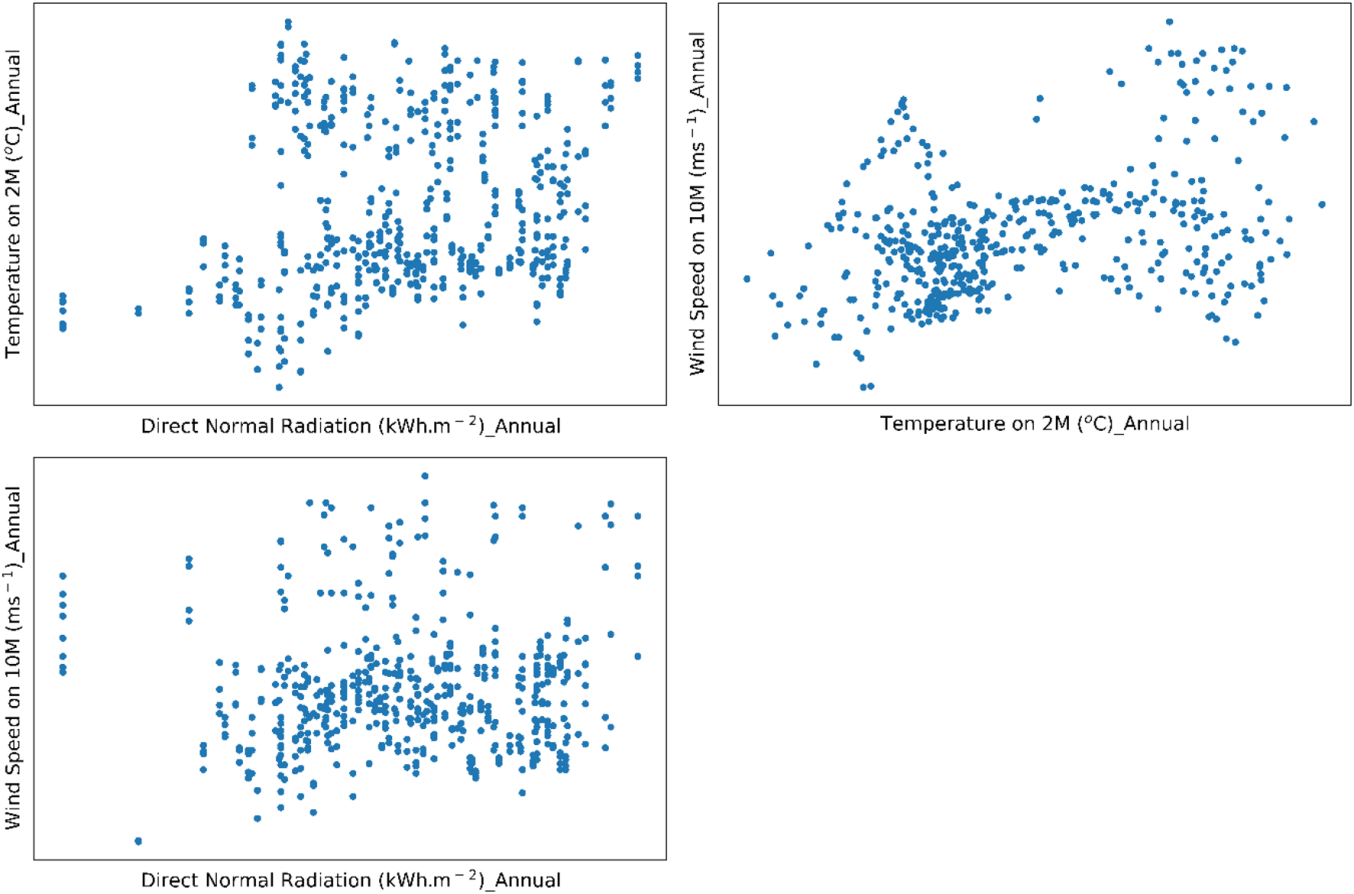
Property-to-property plots are shown for the annual means of temperature in Celsius over 2 m above sea level against the annual means of direct normal radiation in kWh m^−2^ (top left) and against the annual means of wind speed in m/s over 10 m above sea level (top right), and for the annual means of direct normal radiation in kWh m^−2^ against the annual means of wind speed in m/s over 10 m above sea level.

For this purpose, the correlation of annual means (Fig. 1) and seasonal means (Figs. S2, S4, S6 and S8) was evaluated.

Figure 1 shows a positive correlation between the annual mean values of DNR and temperature with a Pearson correlation coefficient at p-value of 0.05 level of 0.21, while a somewhat moderately strong correlation coefficient of 0.37 between the annual mean values of temperature and wind speed shows the dependence on wind speed as the main factor for temperature loss over the Earth's surface. While there is no correlation between DNR and wind speed with a correlation coefficient of 0.06.

However, when evaluating the correlations between parameters across seasons, different behaviours emerge. A moderately strong correlation was found between the mean value of direct normal radiation and temperature, with a correlation coefficient of 0.38, in winter during the period from January to the end of March. The strong correlation between temperature and wind speed with a coefficient of 0.63 shows the strong wind patterns in the winter season and their great influence on the control of temperature fluxes, while no correlation was found between direct normal radiation and wind speed with a coefficient of -0.05.

In spring, in the period from April until the end of June, no correlation was obtained between all the parameters where a correlation coefficient of − 0.06 was obtained between the DNR and the temperature, a coefficient of 0.09 was obtained between wind speed and temperature and a coefficient of 0.021 was obtained between DNR and wind speed.

While in summer, in the period from July to September, a moderate negative correlation between DNR and temperature with a coefficient of − 0.35 show independence of the DNR on increasing the temperature and both parameters might have different impact on the solar ponds’ efficiency especially in high temperature months over the course of the year. Very week correlation was obtained between temperature and wind speed with a coefficient of 0.11 but also no correlation was obtained between DNR and wind speed with a coefficient of − 0.034.

Finally, in autumn in the period between October until the end of December, a moderately positive correlation with a coefficient of 0.147 while a strong positive correlation between temperature and wind speed with a coefficient of 0.61 indicating the high influence of wind speed on the temperature showing the same behaviour as the winter season. A weak positive correlation obtained between DNR and wind speed with a coefficient of 0.13.

Overall, Correlation between climate data shows that there is no correlation between DNR and wind speed during the year. In contrast, there is a moderate correlation between DNR and temperature, which is positive in winter and negative value in summer. The lowest correlation is in the spring, while slightly higher values are seen in the colder autumn months. The strongest correlation is between wind speed and temperature, with peaks in both summer and winter.

**Figure 2:**
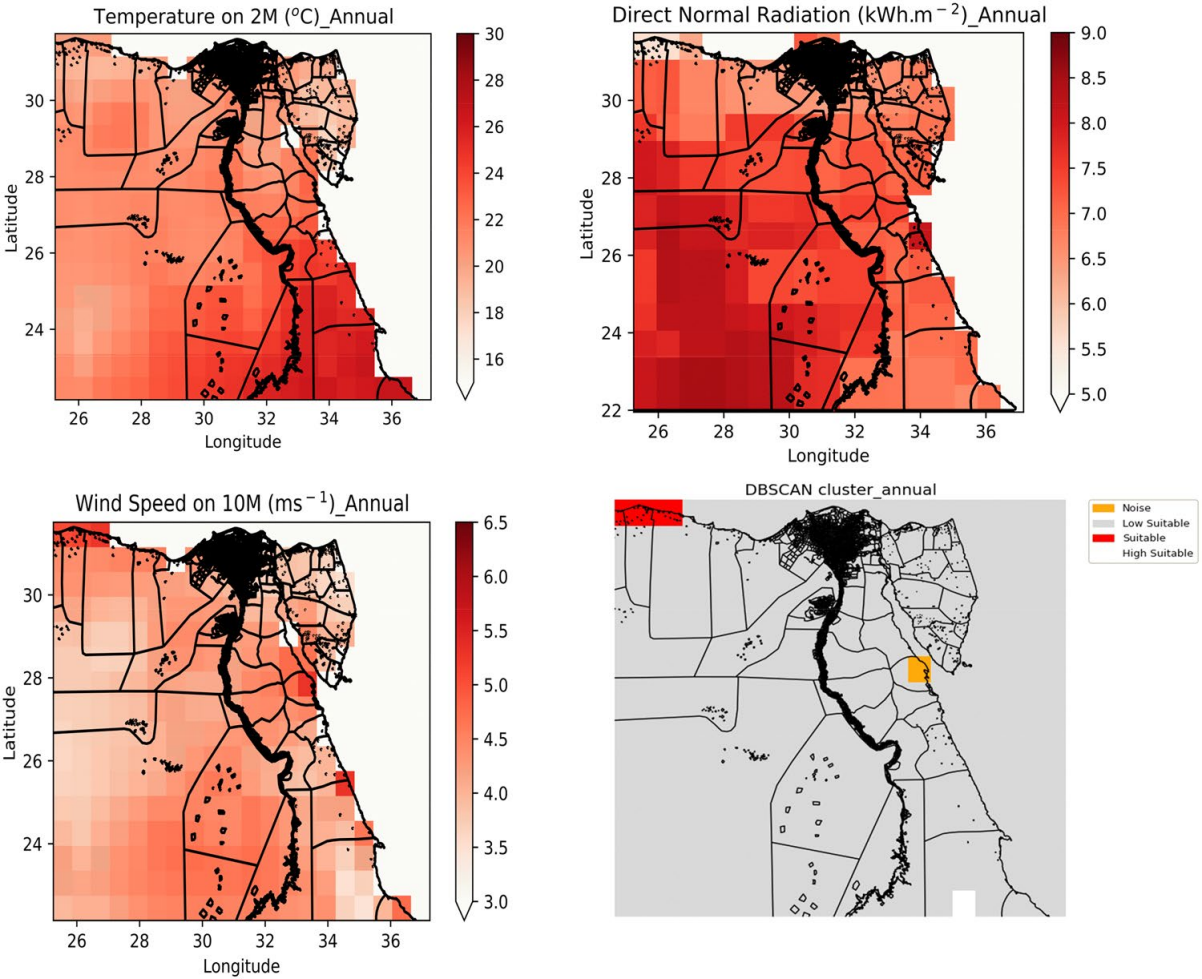
Colormaps display the distribution of annual means of temperature in Celsius for 2 m above sea level throughout Egypt (top left), annual means of direct normal radiation in kWh m^−2^ throughout Egypt (top right), annual means of wind speed in m s^−1^ throughout Egypt (bottom left), and the distribution of DBSCAN clusters obtained from the three annual means. The clusters are categorized as noise (yellow), low suitable (gray), Suitable (red), and high suitable (white). Maps plotted by Python 3.9.13 Spyder environment^30^.

The distribution of annual mean temperature values in Egypt is shown in Fig. 2, top left. The highest (above 28 °C) patterns are shown in the region of latitude from 22° N to 25° N and longitude from 29° E to 36° E. Figure 2, top right shows the distribution of the annual means of DNR in Egypt, where the highest patterns (above 8.5 kWh m^−2^) in a region of latitude between 23° N and 28° N and longitude between 26° E and 30° E. The distribution of the annual means of wind speed is shown in Fig. 2, bottom left where an average of 4.5 ms^−1^ is shown all over Egypt, while few extremes (above 5 ms^−1^) are shown in the Northwestern region close to the Mediterranean Sea.

Similarly, the distribution of climate data by season was assessed. Figure S1 shows an overview of the distribution during the period from January to March. The highest temperature value (above 24 °C) was recorded in a region with a latitude of 26.2° N to 27.7° N and a longitude of 29.3° E to 35° E near the Red Sea. A gradual increase in temperature from 16 to 29 was recorded at the border with Sudan. For DNR, the peak value (over 8 kWh m^−2^) was reached in a region with a latitude of 22° N to 28° N and a longitude of 25.5° E to 29° E. For the wind speed distribution, the peak values (over 6 ms^−1^) were in a region with a latitude of 25° N to 27.8° N and a longitude of 30° E to 35° E near the Red Sea. For the period from April to the end of June (Fig. S3), the distribution of temperature showed the highest value (over 30°C) in a region with a latitude of 22° N to 25.5° N and a longitude of 32.5° E to 34° E. The distribution of DNR showed the peak value (over 9 kWh m^−2^) in a region with a latitude of 22° N to 27.8° N and a longitude of 25.5° E to 29° E. For wind speed, the peak values (above 5.5 ms^−1^) are in a region with a latitude of 25.5° N to 26.5° N and a longitude of 30° E to 35° E in the eastern desert near the Red Sea.

For the period from July to the end of September (Fig. S5), temperature show the maximum values (above 32 °C) in the range of latitude from 22° N to 25° N and longitude from 32.5° E to 34° E and in a tap across the borders of Sudan. For DNR, the distribution shows the peak value (over 9 kWh m^−2^) in an area of latitude 22° N to 29° N and longitude 25.5° E to 30° E in the western desert near the border with Libya. For wind speed, the peak values (over 5.5 ms^−1^) are in an area with latitude 25° N to 27° N and longitude 30° E to 35° E around the Nile Valley in the western desert and continue in the eastern desert to the Red Sea.

For the period from October to the end of December (Fig. S7), temperature shows the peak value (above 24 °C) in a region with latitude from 25.5° N to 27.8° N and longitude from 29.8° E to 35° E. For DNR, the highest values (above 7 kWh m^−2^) in a region with latitude from 22° N to 27.8° N and longitude from 25.5° E to 29° E. For wind speed, the peak values (above 5 ms^−1^) are reached in a region with latitude from 25° N to 27° N and longitude from 30° E to 35° E.

The DBSCAN clustering algorithm was applied for the climate data sets (Temperature, DNR and wind speed) for the annual means and the means of each season (mean of data of three months).

Applying DBSCAN to the data reveals the presence of four main clusters per year (Fig. 2, bottom left). The first cluster, referred to as “noise”, consists of points that are classified as outliers and are not considered part of a cluster. To characterise this noise cluster, a multi-polygon region was created with a latitude between 27.25° N and 28.25° N and a longitude between 33.25° E and 33.75° E. With average values of 7.22 kWh m^−2^, 24.09 °C, and 5.08 ms^−1^ and relative standard deviation (RSD) of 10.7%, 10.9%, and 20.4% for DNR, temperature, and wind speed, respectively, the high RSD values indicate that the cluster is inhomogeneous. The second cluster, which is distributed throughout Egypt, is described as “low suitability”, and has no hotspots. With average values of 5.59 kWh m^−2^, 19.9° C, and 4.8 ms^−1^ and RSD values of 0%, 1.8%, and 6.03% for DNR, temperature, and wind speed, respectively, this cluster shows homogeneity but has somewhat low DNR values. The third cluster is referred to as “suitable”, with an area formed by longitudes between 25.25° N and 26.75° N and latitudes between 31.25° E and 31.75° E. This cluster has average values of 5.59 kWh m^−2^, 19.9° C, and 4.8 ms^−1^, and RSD values of 0%, 1.8%, and 6.03% for DNR, temperature, and wind speed, respectively. Except for the noise cluster, this cluster is homogeneous. The fourth cluster, labelled “very suitable”, includes an area with latitudes between 34.25° E and 34.75° E and longitudes between 21.75° N and 22.75° N where almost the same results were obtained as in the screening carried out in previous work^31^. Its attributes are characterised by an average of 6.8 kWh m^−2^, 27.1 °C, and 5.3 ms^−1^, and RSD values of 0.26%, 1.3%, and 3.4% for DNR, temperature, and wind speed, respectively. Moving from the low suitability cluster to the high suitability cluster, the average value for DNR, the main parameter controlling solar pond performance, increases. DBSCAN clusters were also applied to the data seasonally, with four clusters obtained from January and March data (Fig. S1, bottom right). The “noise” cluster was obtained for the range of latitudes of 27.25° E and 28.75° E and longitudes of 33.35° N and 33.75° N. The cluster was assigned average values of 6.8 kWh m^−2^, 17.9 °C, and 5.07 ms^−1^ and RSD values of 18.3%, 19.2%, and 12.23% for DNR, temperature, and wind speed, respectively. As before, the second cluster is distributed throughout Egypt and is designated as “Low Suitable”. The other cluster is labelled “Suitable” and covers an area with latitudes between 31.25° E and 31.75° E and longitudes between 25.75° N and 27.75° N. The attributes have an average of 4.4 kWh m^−2^, 15.09 °C, and 5.9 ms^−1^ and RSD values of 6.3%, 2.9%, and 4.3% for DNR, temperature, and wind speed, respectively. The last cluster is called “High Suitable” and includes latitudes from 25.75° E to 27.25° E and from 22.25° E to 22.75° E, and longitudes from 30.75° N to 32.25° N and from 34.25° N to 34.75° N. Attributes within the cluster have average values of 6.7 kWh m^−2^, 21.9 °C, and 5.8 ms^−1^ and RSD values of 1.98%, 3.4%, and 4.3% for DNR, temperature, and wind speed, respectively.

Four clusters were identified for the period between April and June (Fig. S3, bottom right), with the first cluster labelled “low suitable” and distributed throughout Egypt. The second cluster is labelled “Moderately Suitable” and includes latitudes from 31.25° E to 31.75° E and longitudes from 25.25° N to 26.75° N. This clusters are characterised by a mean of 6.9 kWh m^−2^, 20.8 °C, and 4.7 ms^−1^, and RSD values of 0.97%, 2.24%, and 3.02% for DNR, temperature, and wind speed, respectively. The other cluster is termed “Suitable” with latitudes from 30.25° E to 30.75° E and with longitudes from 31.25° N to 31.75° N. where this cluster was characterized with attributes with average of 7.5 kWh m^−2^, 24.5° C, and 4.14 ms^−1^, and RSD values of 0%, 1.3%, and 2.8% for DNR, temperature, and wind speed, respectively. The final cluster is designated as “High suitable” and includes latitudes from 23.25° E to 24.25° E and with latitude of 22.75° E and 28.25° E and longitudes from 34.25° N to 34.75° N and with longitude of 35.75° N and 36.25° N. The attributes of the cluster have an average of 7.6 kWh m^−2^, 27.2 °C, and 4.62 ms^−1^ and RSD values of 2.7%, 1.27%, and 2.07% for DNR, temperature, and wind speed, respectively. Four clusters were identified for the period from July to September (Fig. S5, bottom right). Like the results for the last period, the first cluster is identified as “low suitable” and is distributed throughout Egypt. The second cluster is labelled “Moderately suitable” and includes latitudes from 31.25° E to 31.75° E and longitudes from 25.25° N to 26.75° N. The attributes of the cluster have a mean of 7.29 kWh m^−2^, 25.8 °C, and 4.7 ms^−1^, and RSD values of 0.49%, 0.64%, and 3.6% for DNR, temperature, and wind speed, respectively. The third cluster is termed as “Suitable” with latitude from 30.25° E and 30.75° E and longitudes from 31.25° N to 31.75° N. where this cluster is characterized with attributes with mean of 7.5 kWh m^−2^, 28.8 °C, and 3.7 ms^−1^, and RSD values of 0%, 1.03%, and 3.8% for DNR, temperature, and wind speed, respectively. The last cluster labelled “high Suitable”, has attributes with average values of 7.03 kWh m^−2^, 30.3 °C, and 5.02 ms^−1^, and RSD values of 1.2%, 1.4%, and 6.9% for DNR, temperature, and wind speed, respectively. The cluster has latitudes from 22.25° E to 22.75° E and from 23.25° E to 24.25° E and longitudes from 34.25° N to 35.75° N. Looking at both clusters, the last cluster “High Suitable” has a high average temperature in summer, which increases the performance of the solar pond because it can shop more energy. Four clusters were formed for the periods between October and December (Fig. S7, bottom right). The first cluster is referred to as “Noise” where a high deviation was obtained between attributes with average values of 7 kWh m^−2^, 24.5 °C and 4.3 ms^−1^ and RSD values of 11.6%, 8.5% and 9.4% for DNR, temperature and wind speed, respectively. The second cluster is referred to as “Low Suitable” and is distributed throughout Egypt. The third cluster is referred to as “Suitable” and includes latitudes from 31.25° E and longitudes from 23.25° N to 26.75° N. Attributes within the cluster have average values of 3.9 kWh m^−2^, 17.6 °C, and 4.3 ms^−1^ and RSD values of 11.6%, 8.5%, and 9.4% for DNR, temperature, and wind speed, respectively. The last cluster is labelled “Highly Suitable”. The attributes have average values of 6.6 kWh m^−2^, 25.7 °C, and 5.3 ms^−1^, and RSD values of 8.7%, 2.7%, and 6.4% for DNR, temperature, and wind speed, respectively. The cluster has latitudes from 26.75° E to 27.25° E, from 25.75° E to 26.25° E, and from 21.75° E to 22.75° E and longitudes from 34.25° N to 35.75° N, from 29.75° N to 34.25° N, and from 34.25° N to 34.75° N. Throughout the year, an area of longitudes from 25.5°N to 27.5°N and latitudes from 30.5° E to 30.7° E in the northwestern region close to Libya shows suitability (moderate suitable or suitable). This region was consistently identified as suitable during the annual analysis, including the periods between January and March, April and June, July and September, and October and December. Overall, the results of the DBSCAN algorithm yield the clusters where suitability is arranged according to the average values of DNR and the high average values of temperature and the low or constant average values of wind speed. Where the “noise” clusters are assigned according to the higher RSD values.

Where the algorithm results are presented in tabular form where the measured quantities are Epsilon value and Area in km^2^ were mentioned in Table 1. Where DBSCAN was applied for all periods and for annual means with minimum number of samples of three. The epsilon value refers to the radius of the neighbourhood around the data point that the algorithm considers during the process. The area value represents the estimated area suitable for building solar ponds based on the identified cluster.

**Table 1:** DBSCAN clustering epsilon values and areas and locations of high suitable regions for solar ponds.

Time	Epsilon value	Area/km^2^
Annual	0.55	500
January–March	0.64	2000
April–June	0.46	900
July–September	0.58	800
October–December	0.647	3700

The results show that the annual analysis gives an epsilon value of 0.55. For a corresponding area of 500 km^2^, this is the lowest value compared to other values obtained for specific periods of the year. This indicates that the performance of the solar pond is not stable throughout the year. When selecting a site to build a solar pond, it makes more sense to select a specific period. For the period between January and March with an epsilon value of 0.64 and a corresponding area of 2000 km^2^. The results reveal a moderately significant correlation (r = 0.35) between temperature and solar radiation specifically for this period, indicating a meaningful relationship that is not observed in other periods. In situations where such a correlation exists, the solar pond can effectively harness the abundance of solar radiation to generate and store thermal energy, leading to an outstanding performance for heating purposes^32^. This leads to an increase in the conversion efficiency of the solar ponds. In addition, the temperature during this period is optimal, which reduces heat loss and ensures that the energy is effectively stored in the pond. The lowest suitable area for the construction of solar ponds is 900 km^2^ and 800 km^2^ with epsilon values of 0.46 and 0.58 for the period between April and June and between July and September, respectively. During these periods,no significant correlation (r = − 0.06) between temperature and solar radiation between April and June, or when a negative correlation coefficient is observed between July and September, the performance of the solar pond for heating purposes is compromised. In these situations, the solar pond may struggle to capture and utilize the available solar radiation effectively, leading to limited heating capabilities^32^. This is reflected in the DBSCAN algorithm results obtained for the three environmental variables. While for the period between October and December, the highest epsilon value of 0.647 with the highest corresponding area of 3700 km^2^ for the construction of solar ponds. With high solar radiation and energy storage and collection in solar pond.

The application of the DBSCAN algorithm provides several advantages by identifying the best locations for solar pond construction with reasonable accuracy. It also reduces the operational costs of reconnaissance and analysis while accounting for seasonal differences in siting for solar pond construction. The results can aid in the planning and implementation of solar pond projects by utilising resources in the most efficient and effective manner.

In addition, the implementation of the DBSCAN algorithm opens the possibility of considering other factors that affect solar pond performance. Factors such as land use, topography, and soil play an additional role in the optimal location of solar pond projects. Relying on the implementation of DBSCAN for site selection for solar pond development is not the only way used, as it does not consider other factors such as ownership, socioeconomic factors, and infrastructure development.

Overall, the use of the DBSCAN algorithm is very useful in providing an effective and rapid way to locate potential sites for solar pond development and to account for seasonal variations in suitability. However, the algorithm should be used in conjunction with other factors to find the optimal sites for the intended purpose.

## Conclusion

AutoGIS processing in Python provided a simple and effective way to perform site selection for solar pond development. The DBSCAN algorithm was used for clustering analysis of three climate data sets (temperature, direct normal radiation, and wind speed). The model was used for Egyptian boundaries but can be used for other geographic boundaries. A variety of Python libraries were used, including geopandas, osgeo, gdal, rasterio, sklearn,.cluster, rasterio, earthpy, shapely, and matplotlib. The relationships between the three-climate data were evaluated annually and seasonally. Our Python script was used to plot the data in raster images to show the distribution of these parameters both annually and seasonally. The results show that the best sites for solar pond development are in southeastern Egypt and in Northwestern region close to Libya, where site selection varies seasonally. The best time when many sites are available is between October and December with a suitable area of 3700 km^2^. The implementation of AutoGIS processing was helpful for easy processing of remote sensing data. However, the application of DBSCAN in our study is limited by the restriction of important factors such as land characteristics, tenure, socio-economic and infrastructural development. The method can be further improved by consolidating the code into a small platform to facilitate access for users without expertise. However, the DBSCAN algorithm should be implemented in conjunction with other factors to determine the optimal locations for the intended purposes.

## Methodology

**Figure 3:**
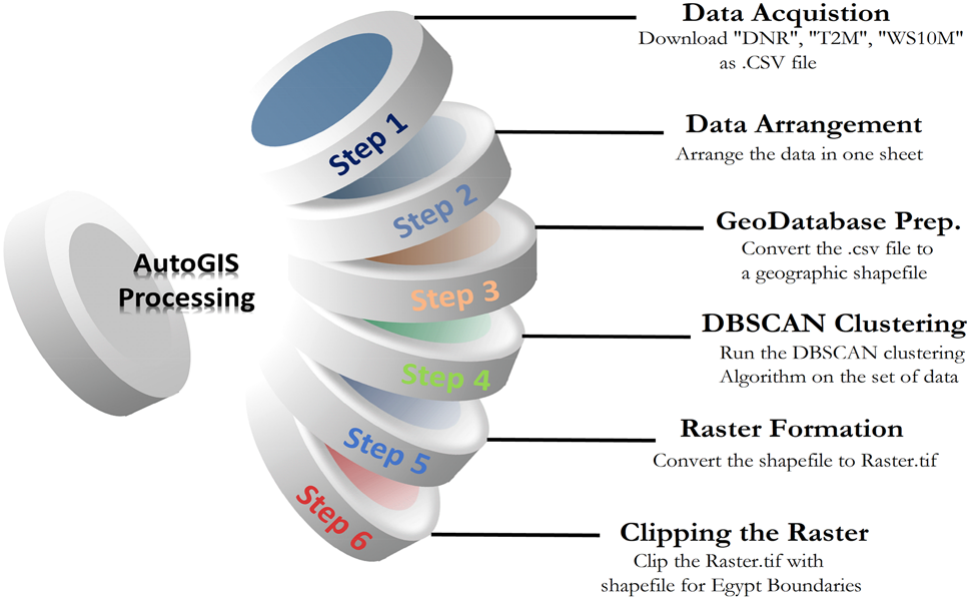
An infographic illustrating the 6-step process of AutoGIS processing using the Python programming language. The process begins with data collection, continues with data processing by arranging the data, preparing the geodatabase, and ends with creating the raster file and clipping the raster with the shapefile for the Egyptian boundaries.

The geospatial data was processed in four main objectives data acquisition (of solar radiation, temperature, and wind speed), geospatial data pre-processing, DBSCAN clustering of the data, and rasterization of the geospatial data while removing the offset geospatial data. Figure 3 shows the illustration of those four objectives is six steps (Data Acquisition (Step 1), Data Arrangement (Step 2), Geodatabase preparation (Step 3), DBSCAN clustering (Step 4), Raster formation (Step 5) and clipping the raster (Step 6)). A short description of each phase is provided in the following subsections.

### Data acquisition

All data were downloaded from the website NASA. Three types of data were collected for this study: solar radiation, temperature at a 2-m height, and wind speed at a 10-m height. Solar radiation data was divided into two variables: direct normal irradiance (DNI) and diffuse horizontal irradiance (DHI). DNI represents the fraction of radiation that directly hits the ground on clear and cloudless days, while DHI represents the fraction of radiation that is scattered as it passes through the atmosphere. For this study, only DNI data was used.

Temperature was measured as the average air temperature (dry bulb) at a 2-m height above the surface. Temperature plays a significant role in the performance of solar ponds, as the water in the pond absorbs solar energy, raising its temperature and supporting power generation and heating. The site’s temperature should be high enough to install a solar pond, and a higher temperature differential leads to higher efficiency.

Wind speed, measured at a 10-m height, is another factor that affects solar pond performance. Wind can increase heat dissipation from the pond’s surface, lowering its temperature and reducing efficiency. Therefore, wind conditions should be considered during solar pond design.

The evaluation of the relationship between DNI, temperature, and wind speed is crucial in selecting a site for solar pond development. These parameters determine the site’s potential for energy production. Historical climate data from 1983 to 2013, obtained from the NASA POWER project, was used to assess the suitability of locations in Egypt for solar pond development. The data was collected over a 30-year period and covered the geographical boundaries of Egypt^33^.

### Geospatial data processing

AutoGIS processing is a powerful tool for displaying and analysing the spatial distribution of environmental parameters such as DNR, temperature, and wind speed. In Python, AutoGIS processing is made even more powerful due to the availability of various libraries such as GDAL, Fiona, Shapely, and Rasterio. These libraries provide a wide range of tools and functions that can be used to handle vector and raster data, making them useful for spatial analysis and data conversion.

The process of AutoGIS processing involves a series of sequential steps. It starts with obtaining the data, which can be done using various methods such as importing a CSV file using the Pandas library^34^. Once the data is acquired, it goes through various processing steps, including data cleaning, filtering, reprojection to a standard coordinate system, and interpolation to create continuous data surfaces.

After the data is processed, it can be displayed using the matplotlib library in Python. This involves converting the shapefile file (vector data) to a raster data format such as a TIFF image, which can then be visualized using a colormap. In the specific case mentioned, the data related to solar radiation, temperature, and wind speed are ordered and arranged in a data frame using the Pandas library. The data is then converted into a geographic shapefile, defining a coordinate reference system (CRS)^35^ using the GeoPandas^36^ and Shapely^37^ and Shapely libraries. These libraries provide support for processing geospatial data types and performing operations on geometric objects.

Overall, AutoGIS processing in Python offers a powerful set of tools and libraries for analyzing and visualizing spatial data, making it a valuable resource for environmental analysis and research.

### DBSCAN clustering

The DBSCAN clustering algorithm was utilized for site selection using three climate datasets. DBSCAN is a widely known technique for identifying clusters and outliers in large spatial datasets. It divides the data into regions of high and low density, based on two sets of values: epsilon, which determines the neighborhood size, and the minimum point value, which determines the number of points required for a dense region. The algorithm starts by randomly selecting an unvisited point and expands the cluster by including all points within the dense region. This process is repeated for each unvisited point.

To apply the DBSCAN algorithm to the data, the GeoPandas library was used to read the data from a shapefile and write the results to a new shapefile. The implementation of the DBSCAN algorithm involved importing the “scikit-learn” library from the cluster module. The data was provided as a 2D array-like object, and hyperparameters such as epsilon (set to 0.55) were adjusted to detect the clusters in the DNR, temperature, and wind speed data. Points within the specified distance were grouped together to form clusters.

### Rasterize the geospatial data

In this part we have carried out two objects. The first object involved rasterizing a shapefile created in the previous part, and the second object entailed clipping a shapefile imported from the Humanitarian Data Exchange website^38^ for the Egyptian borders using the raster obtained in the first object.

To analyze and handle GIS data, the Osgeo package was imported, which provides support for various geospatial data formats. The gdal, osr, and ogr modules from the Osgeo packages were used for processing raster and vector data. The gdal module enables reading and writing geospatial data in raster format, the osr module handles geocoordinate reference systems, and the ogr module deals with reading and writing geodata in vector format.

The code ogr.Open() was used to import the data from the shapefile created previously. A new output raster was created using gdal.GetDriverByName('GTiff').Create(), specifying the pixel size. The extent of the input shapefile was determined, and the number of pixels was calculated.

The Rasterio library was imported to work with and write to the raster datasets. The Fiona library was also imported to read and write geographic data files, specifically reading shape polygons from the specified shapefile. The raster dataset from the first phase was opened with rasterio, and the “mask” function was used to mask the shapefile based on the shape.

### Supplementary Information

Supplementary Information.

## Data Availability

All the data are reported within the manuscript and within the supplementary information.
