# Supplementary material for: AutoGIS processing for site selection for solar pond development as efficient water treatment plants in Egypt
Source: Sci Rep. 2023 Oct 9;13:17009. doi: 10.1038/s41598-023-44047-0 (PMC10562386; doi:10.1038/s41598-023-44047-0)
Supplement: Supplementary file 1 — Supplementary Information. [file 41598_2023_44047_MOESM1_ESM.docx]

# **AutoGIS processing for site selection for solar pond development as efficient water treatment plants in Egypt**

Mahmoud Fatehy Altahan ^1,^*, Mohamed Nower ^2,^*

^1^ Central Laboratory for Environmental Quality Monitoring (CLEQM), National Water Research Center (NWRC), 13621 El-Qanater El-Khairia, Egypt

^2^ Water Management Research Institute (WMRI), National Water Research Center (NWRC), 13621 El-Qanater El-Khairia, Egypt

***** Correspondence: [mahmoud_abdalqader@nwrc.gov.eg](mailto:mahmoud_abdalqader@nwrc.gov.eg) (M.F.Altahan),

mohamed_nower@nwrc.gov.eg (M. Nower)

# **Supplementary Information**


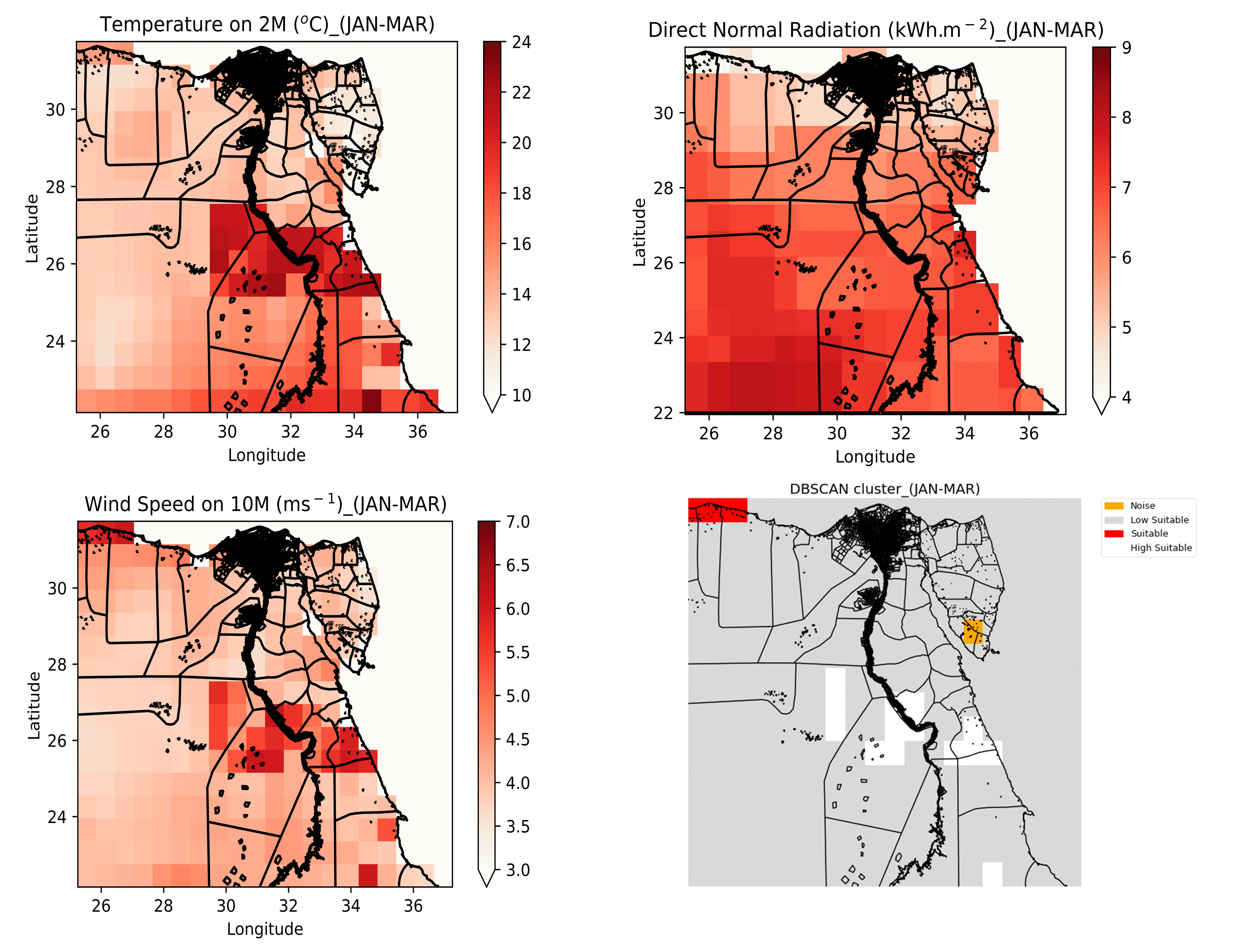


**Figure S1.** Colormaps display the distribution of means of temperature in Celsius for 2 meters above sea level throughout Egypt over the months from January to March (Top Left), annual means of direct normal radiation in kWh.m^-2^ throughout Egypt over the months from January to March (Top Right), annual means of wind speed in m.s^-1^ throughout Egypt over the months from January to March (Bottom Left), and the distribution of DBSCAN clusters obtained from the three means over the months from January to March. The clusters are categorized as Noise (yellow), Low suitable (gray), Suitable (red), and High Suitable (white). Maps plotted by Python 3.9.13 Spyder environment^1^.


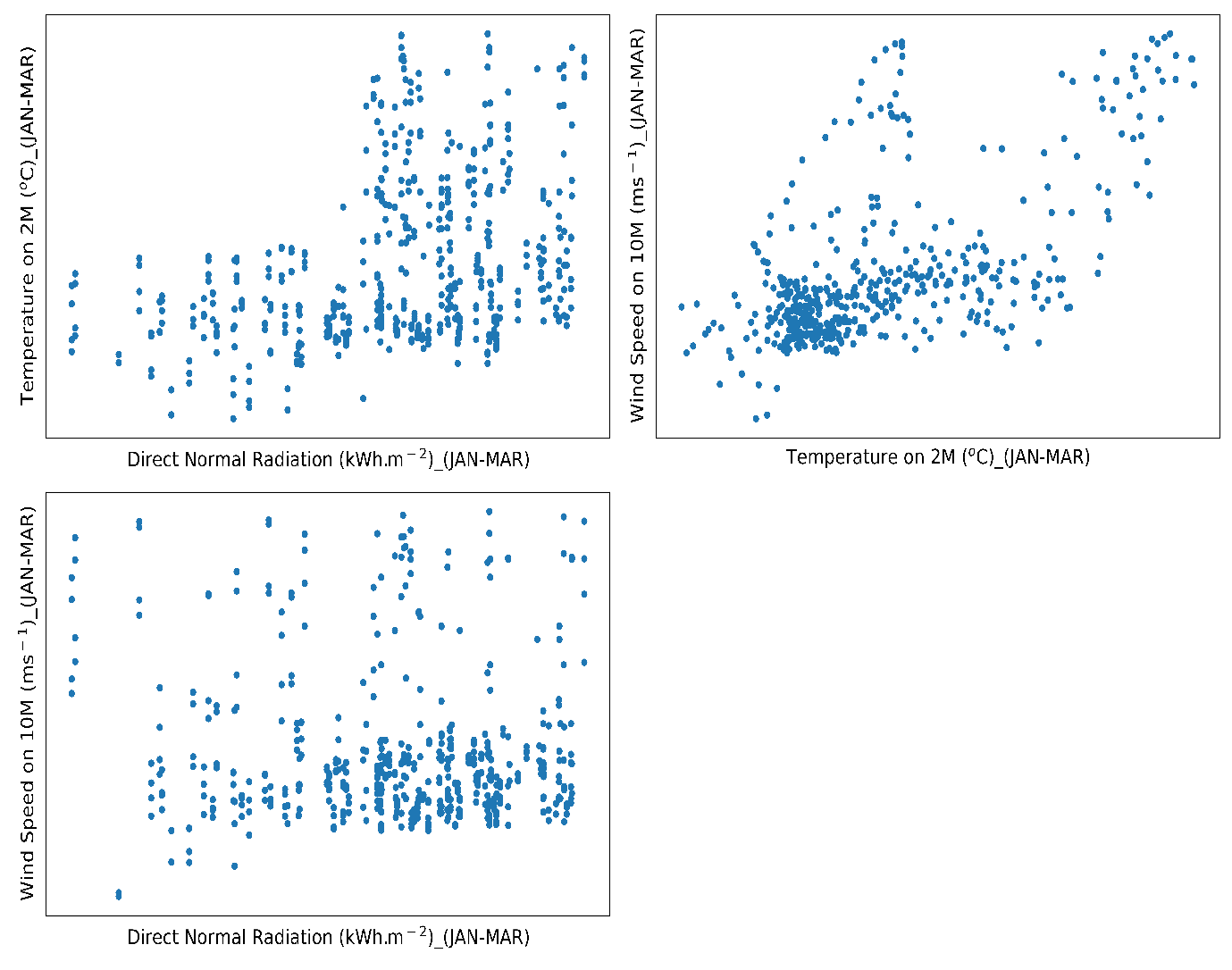


**Figure S2** Property-to-property plots are shown for the means from January to March of temperature in Celsius over 2 meters above sea level against the means from January to March of direct normal radiation in kWh.m^-2^, with a correlation coefficient (Pearson's r = 0.38) (Top Left), for the means from January to March of temperature in Celsius over 2 meters above sea level against the means from January to March of wind speed in m/s over 10 meters above sea level with a correlation coefficient (Pearson's r = 0.63) (Top Right), and for the means from January to March of direct normal radiation in kWh.m^-2^ against the means from January to March of wind speed in m/s over 10 meters above sea level with a correlation coefficient (Pearson's r = -0.05) (Bottom Left).

**
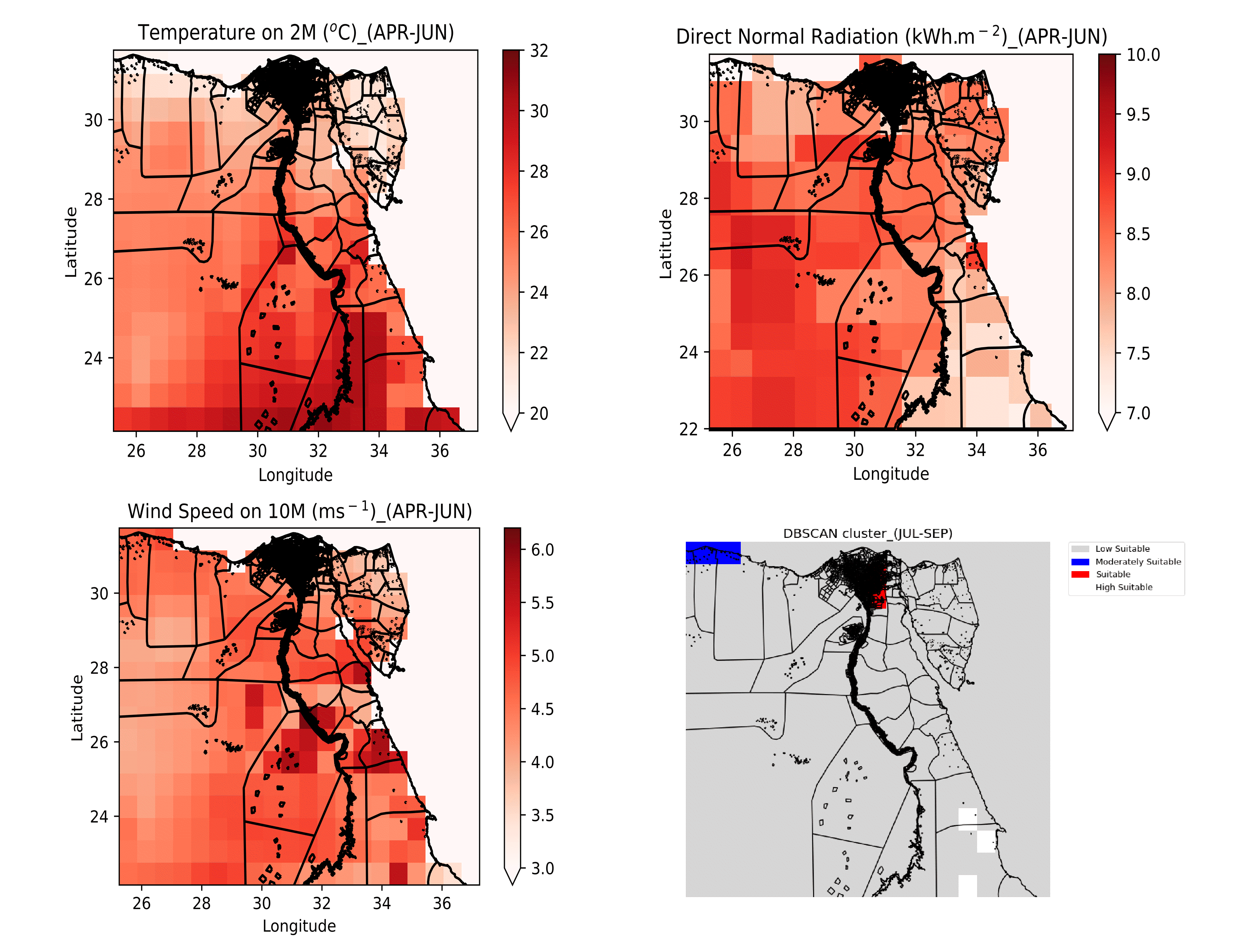
**

**Figure S3.** Colormaps display the distribution of means of temperature in Celsius for 2 meters above sea level throughout Egypt over the months from April to June (Top Left), annual means of direct normal radiation in kWh.m^-2^ throughout Egypt over the months from April to June (Top Right), annual means of wind speed in m.s^-1^ throughout Egypt over the months from April to June (Bottom Left), and the distribution of DBSCAN clusters obtained from the three means over the months from April to June. The clusters are categorized as Low suitable (gray), moderately suitable (blue), Suitable (red) and High Suitable (white). Maps plotted by Python 3.9.13 Spyder environment^1^.


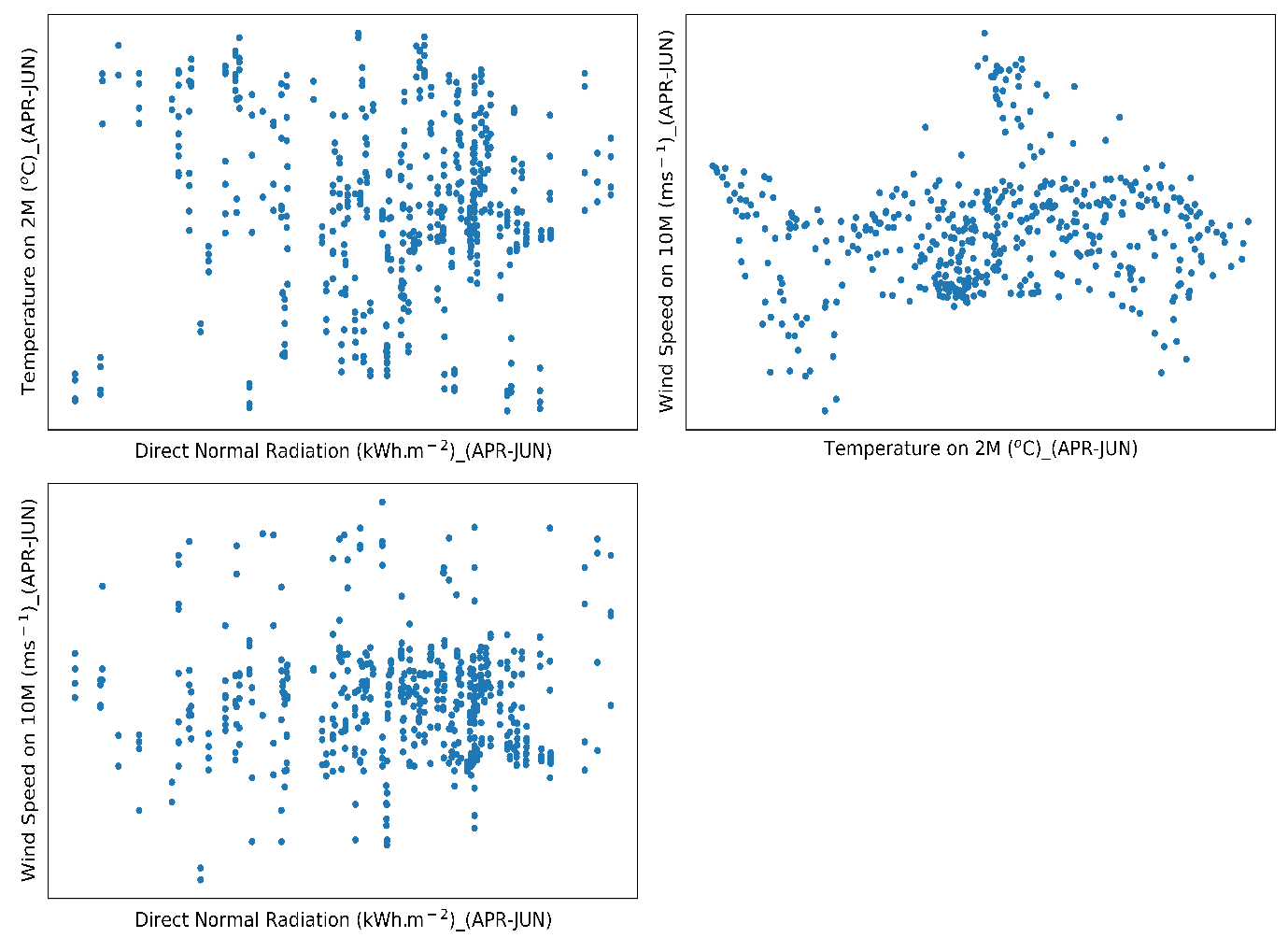


**Figure S4.** Property-to-property plots are shown for the means from April to June of temperature in Celsius over 2 meters above sea level against the means from April to June of direct normal radiation in kWh.m^-2^, with a correlation coefficient (Pearson's r = -0.06) (Top Left), for the means from April to June of temperature in Celsius over 2 meters above sea level against the means from April to June of wind speed in m/s over 10 meters above sea level with a correlation coefficient (Pearson's r = 0.09) (Top Right), and for the means from April to June of direct normal radiation in kWh.m^-2^ against the means from April to June of wind speed in m/s over 10 meters above sea level with a correlation coefficient (Pearson's r = 0.021) (Bottom Left).


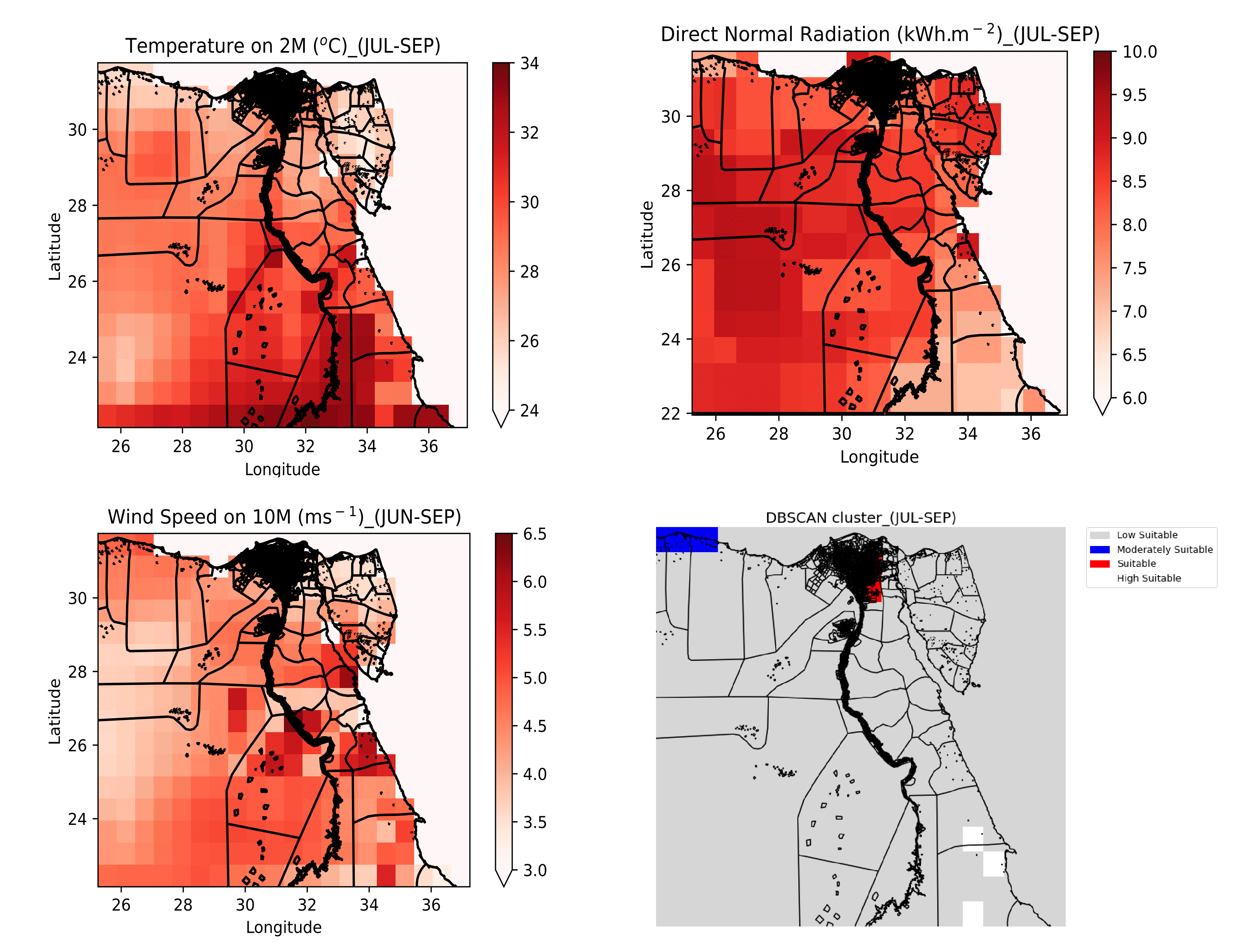


**Figure S5.** Colormaps display the distribution of means of temperature in Celsius for 2 meters above sea level throughout Egypt over the months from July to September (Top Left), annual means of direct normal radiation in kWh.m^-2^ throughout Egypt over the months from July to September (Top Right), annual means of wind speed in m.s^-1^ throughout Egypt over the months from July to September (Bottom Left), and the distribution of DBSCAN clusters obtained from the three means over the months from July to September. The clusters are categorized as Low suitable (gray), Moderately suitable (blue), Suitable (red) and High Suitable (white). Maps plotted by Python 3.9.13 Spyder environment^1^.


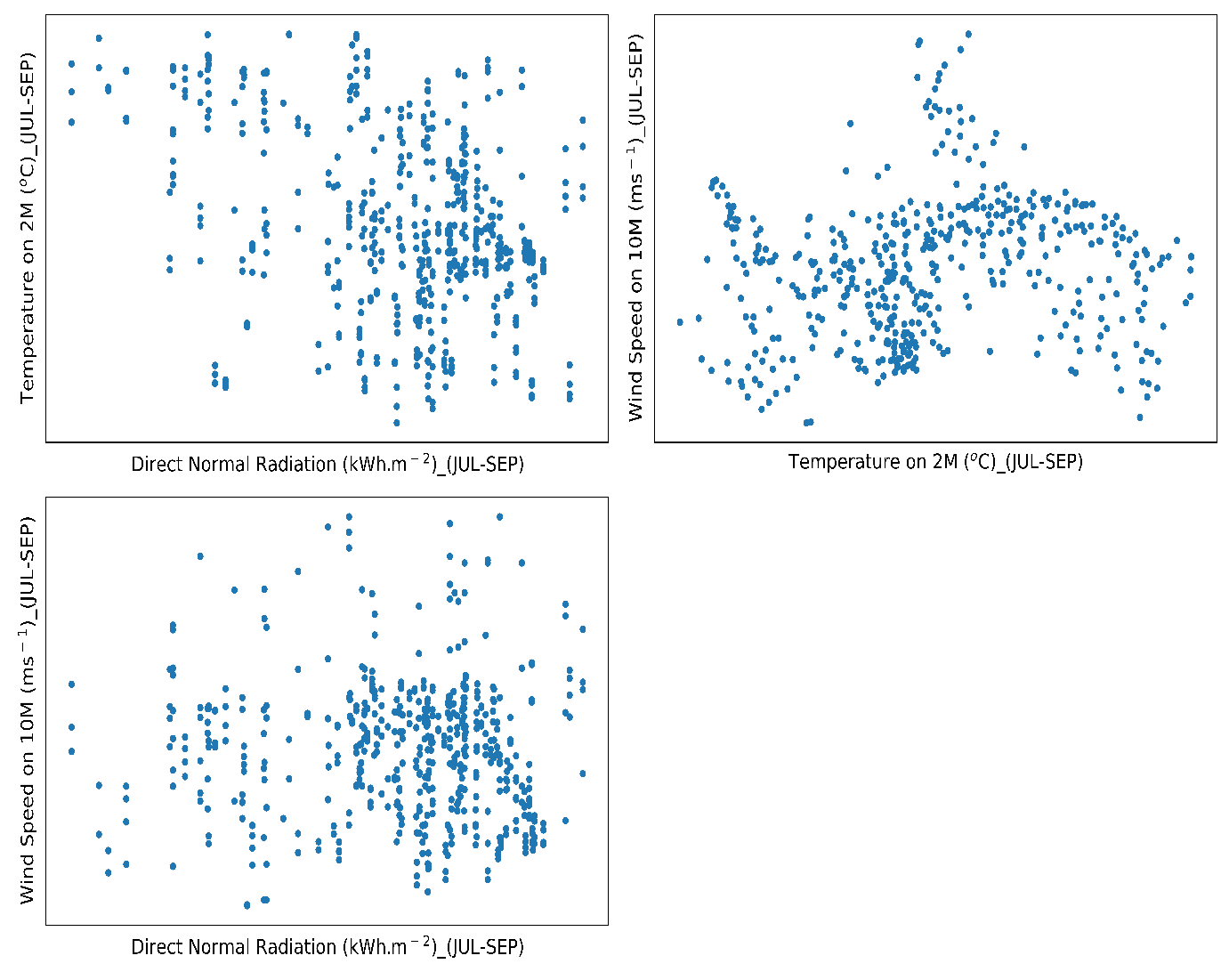


**Figure S6.** Property-to-property plots are shown for the means from July to September of temperature in Celsius over 2 meters above sea level against the means from July to September of direct normal radiation in kWh.m^-2^, with a correlation coefficient (Pearson's r = -0.35) (Top Left), for the means from July to September of temperature in Celsius over 2 meters above sea level against the means from July to September of wind speed in m/s over 10 meters above sea level with a correlation coefficient (Pearson's r = 0.11) (Top Right), and for the means from July to September of direct normal radiation in kWh.m^-2^ against the means from July to September of wind speed in m/s over 10 meters above sea level with a correlation coefficient (Pearson's r = -0.034) (Bottom Left).


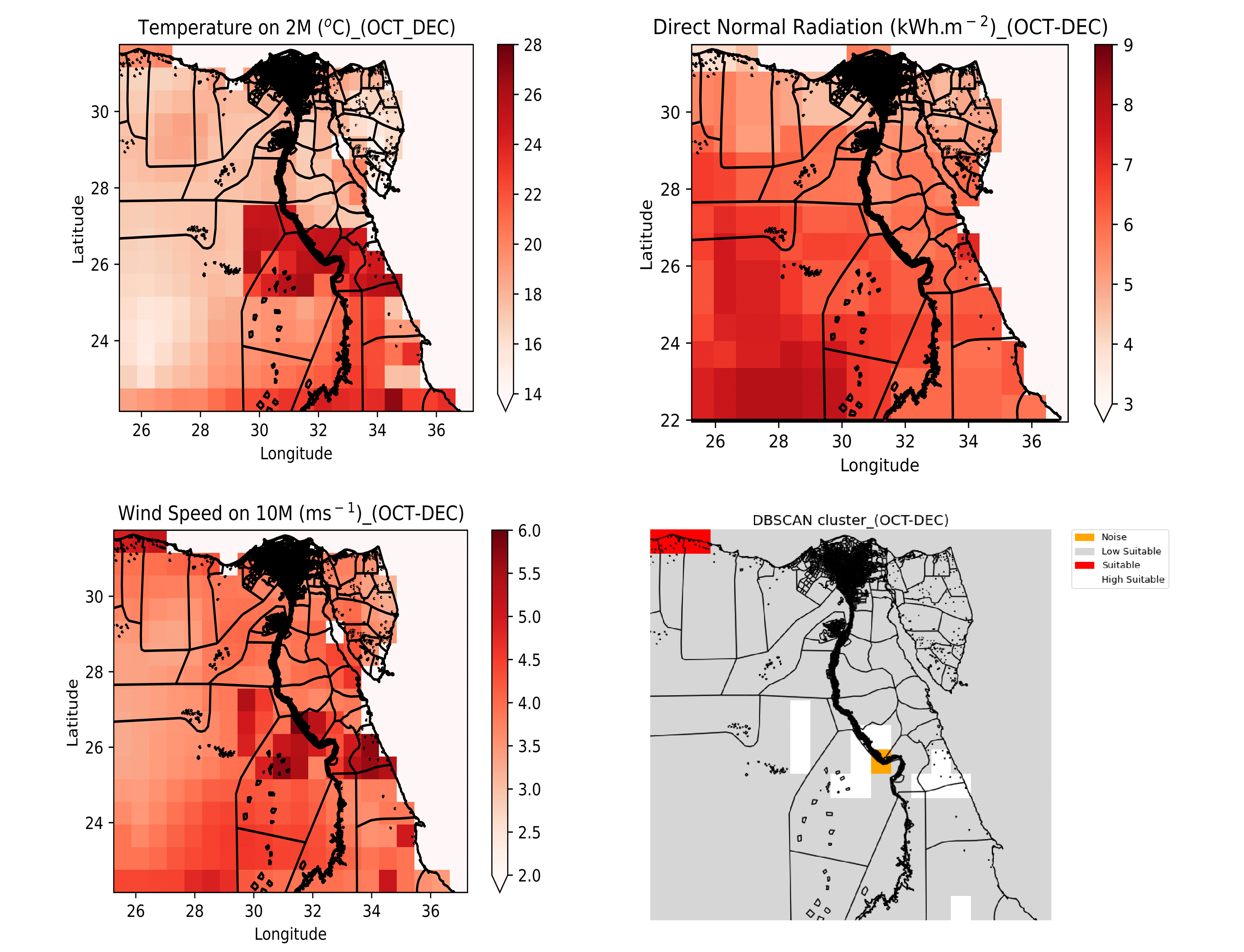
**Figure S7.** Colormaps display the distribution of means of temperature in Celsius for 2 meters above sea level throughout Egypt over the months from October to December (Top Left), annual means of direct normal radiation in kWh.m^-2^ throughout Egypt over the months from October to December (Top Right), annual means of wind speed in m.s^-1^ throughout Egypt over the months from October to December (Bottom Left), and the distribution of DBSCAN clusters obtained from the three means over the months from October to December. The clusters are categorized as Noise (yellow), Low suitable (gray), Suitable (red) and High Suitable (white). Maps plotted by Python 3.9.13 Spyder environment^1^.


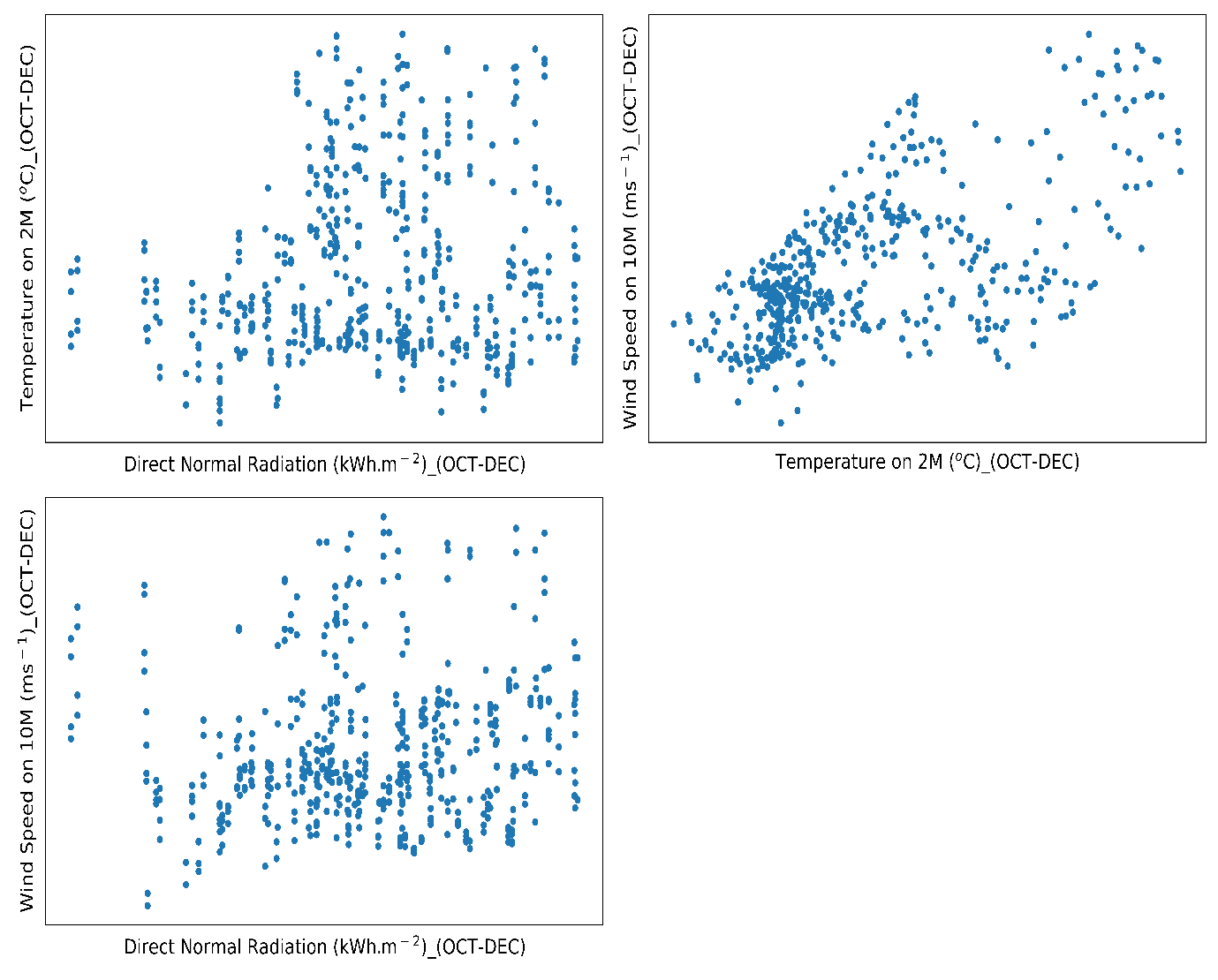


**Figure S8.** Property-to-property plots are shown for the means from October to December of temperature in Celsius over 2 meters above sea level against the means from October to December of direct normal radiation in kWh.m^-2^, with a correlation coefficient (Pearson's r = 0.147) (Top Left), for the means from October to December of temperature in Celsius over 2 meters above sea level against the means from October to December of wind speed in m/s over 10 meters above sea level with a correlation coefficient (Pearson's r = 0.61) (Top Right), and for the means from October to December of direct normal radiation in kWh.m^-2^ against the means from October to December of wind speed in m/s over 10 meters above sea level with a correlation coefficient (Pearson's r = 0.13) (Bottom Left).

## **References**

1 Anaconda, S. *The Scientific Python Development Environment- Python 3.9.13*, <<https://www.spyder-ide.org/>> (Acessed on 20th March 2023).
